# Comparison of conventional and primary sutureless surgery for repairing supracardiac total anomalous pulmonary venous drainage

**DOI:** 10.1186/s13019-019-0853-7

**Published:** 2019-02-08

**Authors:** Yongfeng Zhu, Hewen Qi, Yunzhou Jin

**Affiliations:** 1 Department of Cardiovascular surgery, Zhengzhou Cardiovascular Hospital, Henan cardiovascular disease hospital affiliated to Southern Medical University, Zhengzhou No.7 People’s Hospital, Zhengzhou, 450000 China; 2 Department of Cardiovascular surgery, Tangshan Workers’ Hospital, Tangshan, 063000 Hebei China; 3 Department of Cardiovascular surgery, Chongqing Three Gorges Central Hospital, Chongqing, 404000 China

**Keywords:** Sutureless, Total anomalous pulmonary venous drainage, Mid-term outcomes

## Abstract

**Objective:**

The efficacy of using a sutureless approach in order to surgically manage postoperative pulmonary vein stenosis following total anomalous pulmonary venous drainage (TAPVD) has been reported, though outcomes of primary treatment of supracardiac TAPVD remain unclear. We retrospectively reviewed our cardiac center experience, and compared the differences in mid-term outcomes for those patients that received conventional surgery and those that underwent sutureless technique for the primary repair of supracardiac TAPVD.

**Methods:**

A total of 43 patients (median age, 199 days; range, 35 days to 1572 days) with supracardiac TAPVD underwent surgical treatment at our cardiac center from 2014 to 2018 were studied retrospectively. Primary sutureless repair was conducted in 20 cases (46.5%). The pulmonary vein scores, left ventricular ejection fraction (LVEF), baseline of the included patients, postoperative, and outcomes data were analyzed between the two groups.

**Results:**

The pulmonary vein scores, indicating the stenosis degree, of two groups were 0.1 ± 0.3 and 0.1 ± 0.3, left ventricular ejection fraction (LVEF) (%) were separately 66.2 ± 12.1 and 67.1 ± 13.6. The average cardiopulmonary bypass time of sutureless techniques group was much longer than conventional group (96.2 ± 32.6 min vs 75.6 ± 28.2 min, *P* < 0.05), but there was no difference in aortic cross-clamp time between the two groups. Followed up from 0.1 to 4 years, 3 cases died overall, with 1 (5.0%) individual dying from postoperative pulmonary venous obstruction (PVO) in sutureless group, and 2 (8.6%) dying in the conventional group respectively for postoperative infection and post-PVO. There were no differences in the length of stay in the ICU, grades of PVS after surgery, LVEF and reoperation rate between the two groups.

**Conclusions:**

The mortality, post-PVO, follow up results of supracadiac TAPVD showed no differences between sutureless and conventional techniques. Post-PVO supposed to be the main reason for postoperative mortality.

## Background

Total anomalous pulmonary venous drainage (TAPVD) is an uncommon cardiac malformation wherein no pulmonary veins directly connect to the left atrium, instead connecting only to the right atrium or one of its tributaries. Roughly 45% of TAPVD cases are supracardiac, while one quarter are cardiac, one quarter are infracardiac, and the remaining 5% are of mixed etiology [1–5]. The connection in supracardiac TAPVD is usually to a left vertical vein draining into the left brachiocephalic vein, or more uncommonly into the superior vena cava, where it connects with the right atrium. In rare instances, it may instead connect with the azygos vein.

The most serious complication after TAPVD repair is pulmonary vein obstruction, which may be pulmonary vein intrinsic at the anastomosis. 8 to 54% of cases were reported to occur PVO after operations [6–9]. Comparing the occurrence rate of PVO after operations gradually becomes an important criterion to evaluate the effect of operations.

The general procedure for conducting a sutureless repair of anomalous pulmonary veins is as follows. Initially, a neo-left atrium (LA) is generated via anastomosis of the posterior pericardium to the LA instead of anastomosing the LA directly to the pulmonary venous confluence. This approach has become an increasingly common surgical approach for repairing PVO following TAPVD since it was developed in 1998 [10–12]. Considering effectiveness of this new surgical technique, some researchers had made efforts to apply primary sutureless techniques to TAPVD patients. However, it remains unclear which technique is better. Osami Honjo, Bobby Yanagawa and some other authors compared the two techniques in the repair of TAPVD, however the conclusions were controversial and no consensus have been reached [13, 14].

We hypothesized that the use of such primary sutureless approaches in those patients who have supracardiac TAPVD may allow for better outcomes than conventional surgery, reducing rates of PVO owing to a reduction in confluences. We therefore performed a retrospective analysis of the clinical outcomes for patients over 4 year period that had supracardiac TAPVD, with a particular focus on how primary sutureless technique was related to survival and PVO rates.

## Methods

We performed a retrospective analysis of 43 patients that had undergone surgical repair of supracardiac TAPVD between February 2014 and February 2018. Cardiac, infracardiac and mixed-type TAPVD; single ventricle; associated congenital cardiac lesions, such as right atrial isomerism or hypoplastic left heart syndrome were excluded. All patients were separated based on surgical strategy into two group: 20 cases of sutureless technique group and 23 cases of conventional technique group. The patients were assigned on a rotating basis to 2 experienced cardiac surgeons, including 1 surgeon using the sutureless technique and 1 surgeon using the conventional technique and both cardiac surgeons are experienced in sutureless technique and conventional technique. All the echo exams were performed by an experienced team prior surgery and then during the follow-up period. Table 1 summarizes the baseline characteristics of all patients. Median ages at operation for the two groups were 198 days (range, 35–1530 days), 202 days (range,42–1572 days) and median weights were 7.3 kg(3.2–12.8), 7.1 kg(3.4–13.2). The median follow-up duration was 2.9 years (range, 0.1 to 4) and 2.8 years (range, 0.1 to 3.8).

**Table 1:** Baseline characteristics and operative data of the supracardiacTAPVC patients

Variables	Sutureless group (20)	Conventional group (23)	*P* value
Age(d)	198(35–1530)	202(42–1572)	0.843
Weight(kg)	7.3 (3.2–12.8)	7.1(3.4–13.2)	0.833
Male (%)	10(50%)	11(47.8%)	0.886
LVEF (%)	66.2 ± 12.1	67.1 ± 13.6	0.821
PVS	0.1 ± 0.3	0.1 ± 0.3	0.99
CPB(min)	96.2 ± 32.6	75.6 ± 28.2	0.031*
aortic cross-clamp time time	62.8 ± 22.4	58.9 ± 19.8	0.547
DHCA(%)	2(10%)	0(0%)	0.12

*LVEF* Left ventricular ejection fraction, *PVS* pulmonary vein stenosis, *CPB* cardiopulmonary bypass, *DHCA* deep hypothermic circulatory arrest. *P < .05

### Pulmonary vein score

The pulmonary vein score for each individual vein, which used to evaluate the anomaly pulmonary vein before surgical intervene, was calculated as Yun TJ reported. Briefly, pre- and postoperative echocardiographic data were reviewed to quantify the degree of PVO: 0 = no stenosis (mean gradient < 2 mmHg); 1 = mild stenosis (mean gradient 2.0–6.9 mmHg); 2 = severe stenosis (mean gradient > 7 mmHg); and 3 = complete occlusion^11^. The sum of the individual pulmonary vein scores is then used as a subjective measure of the overall degree of PVO. PVS was assessed 0.2 ± 0.5 in two groups andshowed no statistic differences.

### Surgical technique

**Fig. 1:**
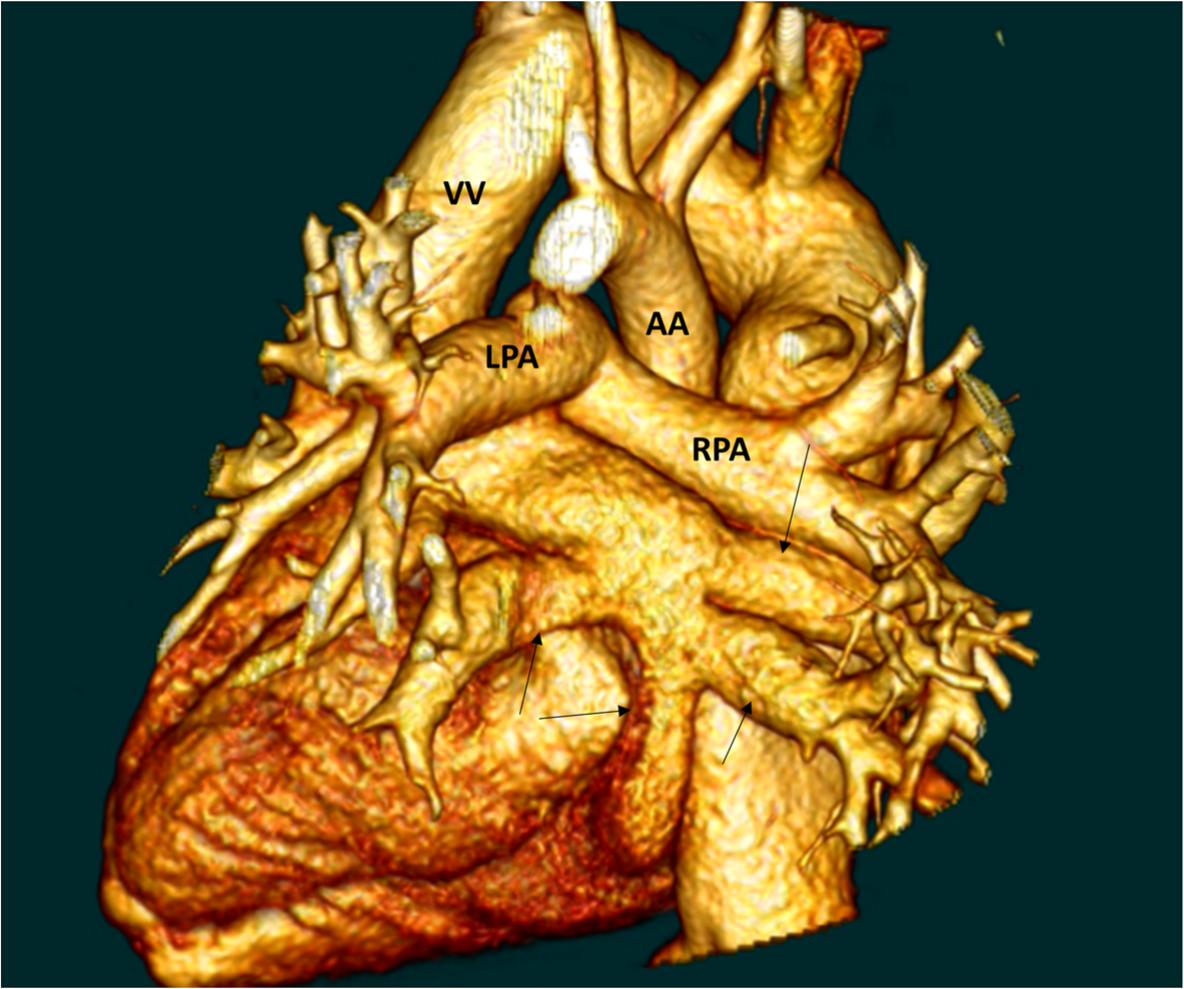
**a** A parallel incision was made on the dome of the left atrium beginning at the base of the left atrial appendage and another transverse incision was made at the common pulmonary venous confluence.**b** Left atrium and the posterior pericardium were then anastomosed as a continuous suture after ligation of the vertical vein

For supracardiac TAPVD, after ligation of the vertical vein at the level of the innominate vein. The superior vena cava, ascending aorta and pulmonary artery were retracted laterally to expose the dome of the left atrium and the common pulmonary vein. A parallel incision was made on the dome of the left atrium beginning at the base of the left atrial appendage and another transverse incision was made at the common pulmonary venous confluence. The common pulmonary vein was then anastomosed to the left atrium. A pericardial patch was used for atrial septal defect (ASD) closure. For sutureless group, as Bobby Yanagawa et al. reported, the venous confluence was initially cut and the incisions were extended onto each individual pulmonary vein [14]. This cut was then further extended to the pleural-pericardial reflection laterally. The LA and the posterior pericardium were then anastomosed as a continuous suture (Fig. 1).

### Statistical analysis

Continuous variables were expressed as mean and standard deviation (SD) or as median and range. Comparison of dichotomous variables was performed using the χ2 test or two-tailed Fisher’s exact test. Statistical analyses were performed using SAS (Version 9.1; SAS Institute Inc., Cary, NC) and R (Version 2.10, R Project for Statistical Computing). A two-tailed *P*-value ≤0.05 was considered statistically significant.

## Results

**Fig. 2:**
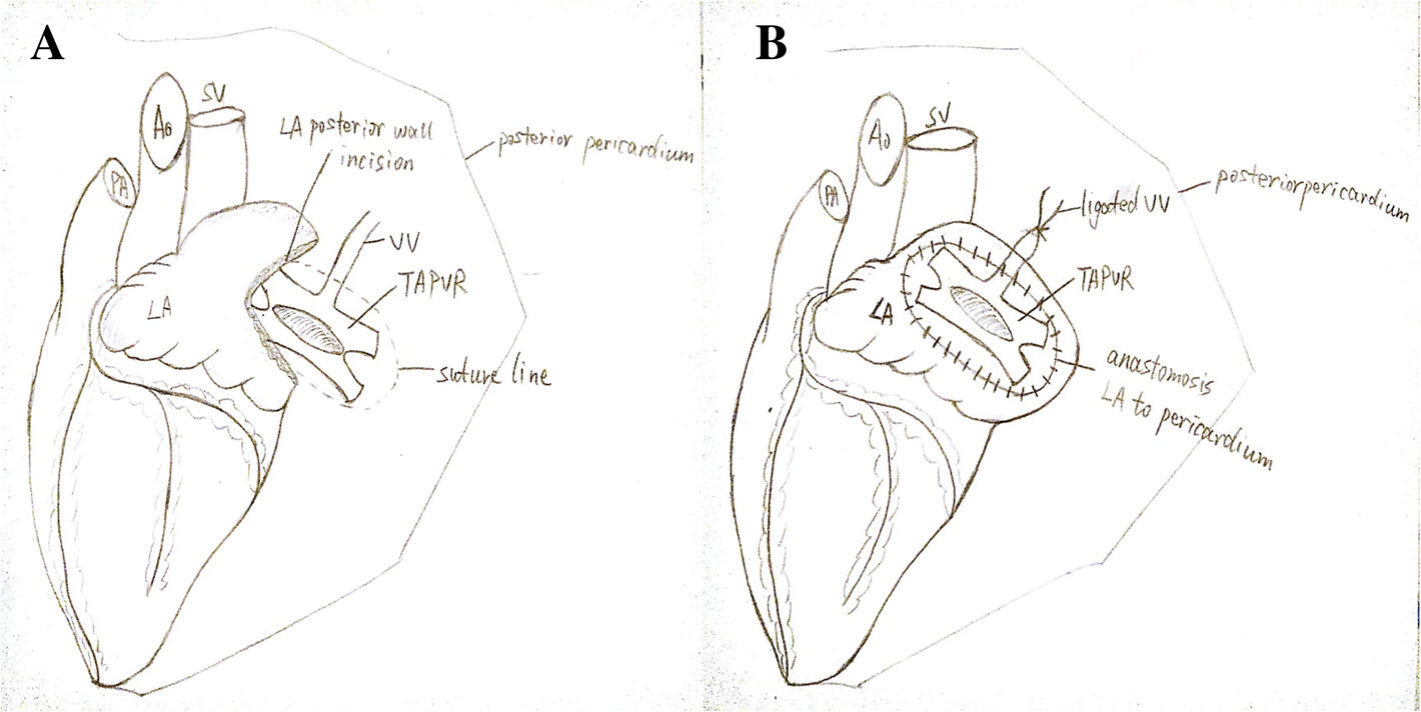
Three-dimensional CT showed the supracardiac TAPVD. Black arrow: pulmonary vein; VV, Vertical vein; AA, Ascending aorta; LPA, Left pulmonary artery; RPA, Right pulmonary artery

A total of 43 patients diagnosed with supracardiac TAPVD formed the study cohort (Fig. 2). Sutureless techniques were performed on 20 patients and conventional on the other 23 patients. PVS was evaluated 0.1 ± 0.3 in sutureless group, 0.1 ± 0.3 in conventional group, showing no statistic differences. Sex, age, weight, LVEF were comparable between these two groups (Table 1).

Aortic cross-clamp time was similar in both groups. However, the sutureless group had a longer average total cardiopulmonary bypass (CBP) time (96.2 ± 32.6 min vs 75.6 ± 28.2 min, *P* < 0.05). This longer CBP times for was most likely a result of the complex anatomic structures and it may took more time to separate and anastomose in sutureless groups. Deep hypothermic circulatory arrest was performed on 2 patients (10%) in sutureless group because of much pulmonary venous return to the sutureless site affected the anastomosis achieve. (Table 2).

**Table 2:** Postoperative, and outcomes data of the supracardiac TAPVC patients

Variables	Sutureless group (20)	Conventional group (23)	*P* value
Length of ICU stay (d)	11(5–41)	12(5–45)	0.516
PVS	0.2 ± 0.5	0.2 ± 0.5	0.99
LVEF (%)	68.6 ± 13.4	70.4 ± 12.9	0.656
Reoperation	1(5%)	1(4.3%)	0.919
Cardiac death	1(5%)	1(4.3%)	0.919
Noncardiac death	0(0%)	1(4.3%)	0.34
Follow-up time (year)	2.9(0.1–4)	2.8(0.1–3.8)	

*PVS* pulmonary vein stenosis, *LVEF* Left ventricular ejection fraction

There were no significant differences in postoperative characteristics between groups. PVS in two groups were both 0.2 ± 0.5, postoperative LVEF (%) showed no differences among two groups (68.6 ± 13.4 vs 70.4 ± 12.9, *P* > 0.05). Average length of ICU stay of sutureless group was 11 days (range 5 to 41 days), 12 days (range 5 to 45 days) in conventional group, there were no statistic differences (Table 2).

Patients were followed for a median of 2.9 (0.1–4) years for those undergoing conventional surgery and 2.8(0.1–3.8) years for those that underwent the sutureless technique. One patient in the sutureless group needed to undergo a second operation to treat PVO (5%) and survived to the last follow up without PVO, The same one in the conventional repair group (4.8%). One patient died in sutureless group for postoperative PVO (5%) two months after the surgery, one died in conventional group for postoperative infection (4.8%) one week after the surgery and another died in conventional group for postoperative PVO (4.8%) two weeks after the surgery. Overall mortality or freedom from reoperation did not differ significantly between these two groups.

## Discussion

Although initially employed as a means of treating postoperative PVO, sutureless approaches have been more recently utilized for primary TAPVD repair in high risk patients like to develop PVO following the repair operation, with the goal of improving their long-term survival. Patients at particular risk of PVO include those who have preoperative hypoplastic pulmonary veins, are of a young age at the time of the initial surgery, as well as those who have TAPVD with right atrial isomerism, cardiac TAPVD with preexisting PVO, or mixed TAPVD [15–18]. A potential advantage of sutureless technique is a more limited reactive intimal proliferation because the suture line is not directly on the pulmonary vein. There are also advantages with respect to no direct suture line distortion or narrowing of the veins, particularly if they are small. Optimal flow characteristics for a given vein are therefore intact.Following conventional surgery, post-operative PVO can develop as a consequence of either fibrosis or inflammation at the site of the suture between LA and pulmonary veins, besides, mismatching of anastomotic confluent between LA and pulmonary vein may be the cause of post-PVO, sutureless technique allowed the aggressive resection of the obstructed pulmonary veins tissue and avoided surgically induced distortion of the pulmonary veins, which may help to prevent subsequent PVO.

Yoshimura et al. have suggested that the use of the sutureless approach may be less technical than the conventional surgical approach [19] A potential advantage of sutureless technique is a more limited reactive intimal proliferation because the suture line is not directly on the pulmonary vein. There are also advantages with respect to no direct suture line distortion or narrowing of the veins, particularly if they are small. Optimal flow characteristics for a given vein are therefore intact. As a result, the suretureless technique is supposed to call for less CPB and aortic cross-clamp time. However, we found no significant difference on CPB time or aortic cross-clamp time, and indeed on the contrary the average CPB time was longer than conventional technique (96.2 ± 32.6 min vs 75.6 ± 28.2 min, *P* < 0.05). The longer cross clamp and deep hypothermic circulatory arrest times for sutureless repair were most likely related to surgeon bias because the surgeon who performed some sutureless repairs chose to do so with circulatory arrest. Surgical complexity or unstable situation of patients may be another reason for longer CPB time. ICU stays are related to the post-operative recovery, there were no significant differences between two groups. The total hospitalization time is mainly determined by ICU management and accurate preoperative diagnosis, so standard ICU management and diagnosis should be conducted during perioperative period.

Mauro Lo Rito et al. analyzed 195 patients who underwent TAPVD repair during 1990 to 2012 and concluded that there was a lower PVO incidence in their primary sutureless group relative to their standard repair group, particularly in those TAPVD patients of the infracardiac or mixed type, with a lower pulmonary vein score [20]. Bobby Yanagawa et al. enrolled 57 patients TAPVD repair during 1997 to 2009, and come to a conclusion that sutureless technique is a useful technique for surgeons to employ, particularly in complicated cases, including infracardiac TAPVD [14]. Our results showed no significant differences in mortality and post PVO between two groups in supracardiac TAPVD, Ages at operation in our research were about 200 days old. However, age at operation of TAPVD is neonate or early infant. That may be associated with our encouraging results.

## Conclusions

There are no significant differences in mortality and post PVO between sutureless technique group and conventional technique group in supracardiac TAPVD patients. Sutureless technique can be used for supracardiac TAPVC with good results. The main reason for postoperative mortality is Post-PVO.

## Abbreviations

AA: Ascending aorta
CPB: Cardiopulmonary bypass
DHCA: Deep hypothermic circulatory arrest
LA: Left atrium
LPA: Left pulmonary artery
LVEF: Left ventricular ejection fraction
Post-PVO: Post pulmonary venous obstruction
PVO: Pulmonary venous obstruction
PVS: Pulmonary vein stenosis
RPA: Right pulmonaryartery
SD: Standard deviation
TAPVD: Total anomalous pulmonary venous drainage
VV: Vertical vein
